# Prolonged Retention of Life by Infants Who Have Not Breathed

**Published:** 1868-10-01

**Authors:** James T. Newman


### PROLONGED RETENTION OF LIFE BY INFANTS WHO HAVE NOT BREATHED.

BY JAMES T. NEWMAN, M.D.

AUGUST the 14th, 1868, was called to see a young woman who had recently given birth to a child, the attendants becoming alarmed at the amount of blood the girl was losing. Had not this been the case, in all human probability, the subject of which I am about to relate would never have been presented to your consideration. At half-past twelve on the evening above mentioned, I visited the patient and found her suffering from hæmorrhage. I immediately took a couple of napkins, folded them, making a compress, placing them at the lower part of the abdomen, so as to prevent the womb from filling with blood. The woman recovered; but the interest of this paper does not rest upon some particular method of treatment, but in a very singular phenomenon, and one wholly new to me, but in searching some of the French and German authors, I find that Solomon was right when he said there was nothing new under the sun.

The friends of the girl were doing all in their power to conceal her shame, and had they not despaired of her life, none but the family would ever have known what the matter was. In making this known I feel confident that I am committing no breach of honor. The child under consideration was born at eight in the morning, and was quietly wrapped up in an old blanket and put out of sight. I was told that it was still-born. I do not know why that I requested to see it, but suffice it to say that the child was shown me, and there was something in its face told me that it was not dead; but I said nothing. The next morning I had an occasion to use the stethoscope on an old lady living in the same locality. I called in to see my patient. After finding her doing well, I asked to see the child and was told that it was in the coffin. I still looked as if I would like to see it, and the mother noticing my countenance, raised the lid. I took the stethoscope and placed it over the region of the heart, and to my great astonishment I could distinctly hear the sound of the heart. I took the child out of the coffin, used Marshal Hall method. In the course of thirty minutes the child commenced breathing; the pulse was natural; it cried, and took the breast eagerly. It is a fine looking boy to-day, and for aught I know, bids fair to live three score and ten years. Since seeing this very remarkable case, there is no doubt in my mind that many children are consigned to the grave without an effort to induce respiration.

